# Psychedelic-Induced Serotonin 2A Receptor Downregulation Does Not Predict Swim Stress Coping in Mice

**DOI:** 10.3390/ijms232315284

**Published:** 2022-12-04

**Authors:** Błażej D. Pędzich, Mireia Medrano, An Buckinx, Ilse Smolders, Dimitri De Bundel

**Affiliations:** Research Group Experimental Pharmacology, Department of Pharmaceutical and Pharmacological Sciences, Center for Neurosciences, Vrije Universiteit Brussel, 1090 Brussels, Belgium

**Keywords:** behavioral despair, 5-HT2A, downregulation, DOI, psychedelics, stress, mPFC, head twitch, mice

## Abstract

Serotoninergic psychedelics such as psilocybin have been reported to elicit a long-lasting reduction in depressive symptoms. Although the main target for serotoninergic psychedelics, serotonin type 2A receptor (5-HT_2A_), has been established, the possible mechanism of the antidepressant action of psychedelics remains unknown. Using the mouse forced swim test model, we examined whether the administration of the synthetic serotoninergic psychedelic 2,5-dimethoxy-4-iodoamphetamine (DOI) would modulate 5-HT_2A_ receptor levels in the medial prefrontal cortex (mPFC) and revert stress-induced changes in behavior. Mice subjected to swim stress developed a passive stress-coping strategy when tested in the forced swim test 6 days later. This change in behavior was not associated with the hypothesized increase in 5-HT_2A_ receptor-dependent head twitch behaviors or consistent changes in 5-HT_2A_ receptor levels in the mPFC. When DOI was administered 1 day before the forced swim test, a low dose (0.2 mg/kg i.p.) unexpectedly increased immobility while a high dose (2 mg/kg i.p.) had no significant effect on immobility. Nevertheless, DOI evoked a dose-dependent decrease in 5-HT_2A_ levels in the mPFC of mice previously exposed to swim stress. Our findings do not support the hypothesis that the downregulation of 5-HT_2A_ receptors in the mPFC contributes to the antidepressant-like properties of serotoninergic psychedelics.

## 1. Introduction

Major depressive disorder is a common and severe mental disorder that affects more than 300 million people globally. It is the leading cause of disability, a major contributor to the global burden of disease and an important cause of age-standardized life-years lost [1,2]. The available evidence indicates that the majority of individuals with major depressive disorder do not achieve and sustain full remission with the currently approved antidepressant treatments [2,3]. Psychedelic substances have been used for spiritual and medicinal purposes for thousands of years [4], and emerging clinical evidence supports their use for the treatment of psychiatric disorders [5]. Recent studies indicate that treatment with serotoninergic psychedelic psilocybin can induce a rapid-onset and long-lasting decrease in symptom severity in patients suffering from treatment-resistant depression [6,7,8,9,10]. However, it is a subject of active debate as to what extent the acute psychedelic response contributes to the observed therapeutic effects of serotoninergic psychedelics [11,12].

Serotoninergic psychedelics, such as psilocybin, mescaline, dimethyltryptamine (DMT) or lysergic acid diethylamide (LSD) exert their effects on altered consciousness and positive mood states through the activation of serotonin (5-hydroxytryptamine, 5-HT) receptors. Most notably, they act as biased (partial or full) agonists of 5-HT_2A_ receptors, and this is widely considered to underlie their psychedelic effects in humans [13,14,15,16,17,18]. Nevertheless, serotoninergic psychedelics may also bind to other receptors such as 5-HT_2C_ and 5-HT_1A_ receptors [19,20,21,22,23,24,25,26]. Interestingly the 5-HT_2A_ receptor is also a common validated target of several classes of psychotropic drugs such as atypical antipsychotics, that are effective against negative symptoms of schizophrenia, but also a target of several clinically used antidepressants, such as trazodone and mirtazapine [27,28,29]. However, these drugs act as antagonists of the 5-HT_2A_ receptor. Nevertheless, the notion that 5-HT_2A_ agonism is an undesirable property for psychotropic medication has recently been challenged [5].

Serotoninergic psychedelics induce 5-HT_2A_ receptor internalization from the plasma membrane to the cytoplasm [30,31,32]. Paradoxically, atypical antipsychotics that act as 5-HT_2A_ receptor antagonists also elicit 5-HT_2A_ receptor internalization [33,34]. Interestingly, the tethering of 5-HT_2A_ receptors to the cell membrane by a post-synaptic density of 95 kDA (PSD-95) appears critical for the acute effects of both psychedelics and atypical antipsychotics [35]. The downregulation of 5-HT_2A_ receptors has been observed following the chronic administration of selective serotonin receptor inhibitors (SSRI) [36,37,38,39], and the genetic disruption of 5-HT_2A_ signaling was shown to interfere with the antidepressant action of SSRI [40]. This raises the question of whether the downregulation of 5-HT_2A_ receptors and the subsequent changes in 5-HT_2A_ signaling may contribute to antidepressant drug action. In support of this notion, 5-HT_2A_ antagonism exerts antidepressant-like effects [41,42,43,44] and potentiates the antidepressant-like effects of SSRIs and other antidepressants [40,42,45,46,47]. Moreover, atypical antipsychotics are clinically effective in augmenting SSRI treatment in treatment-resistant depression [48,49].

Studies on 5-HT_2A_ receptor levels in patients suffering from depression show inconsistent results. While most post-mortem studies find increased 5-HT_2A_ receptor levels in the prefrontal cortex of suicide victims with depression [50,51,52,53,54,55], some studies report no difference [56,57]. While a handful of positron emission tomography (PET) studies found increased 5-HT_2A_ receptor binding in healthy subjects at risk of depression [58,59], medication-free recovered patients [60] and severely depressed patients [61], most PET studies found evidence for decreased prefrontal cortex 5-HT_2A_ receptor expression in subjects with depression [62,63,64,65,66]. One potential confounding factor is that the treatment of patients with SSRI may also decrease 5-HT2A receptor levels [67,68,69]. In rodents, stress exposure was previously reported to induce a delayed and long-lasting increase in the density of 5-HT_2A_ receptors in the frontal cortex [70,71,72,73,74,75], which is associated with the augmentation of 5-HT_2A_ receptor-mediated head twitch responses [71,76,77,78,79,80]. Similar increases in 5-HT_2A_ receptor expression and sensitivity were observed following the chronic stimulation of glucocorticoid release [81] or increased glucocorticoid receptor activation [82,83]. This suggests that 5-HT_2A_ receptor expression is under glucocorticoid receptor control [84]. Nevertheless, some studies also found no significant effects of stress on cortical 5-HT_2A_ receptor binding or 5-HT_2A_ receptor-mediated head twitch responses in rodents [85,86,87]. The effect may depend on the type and duration of stress exposure [88].

Several previous studies have shown that serotoninergic psychedelics can decrease immobility in rodents during the forced swim test, which is typically interpreted as an antidepressant-like effect [89,90,91]. These effects may be dependent on prior exposure to stress and are typically long-lasting [90,91]. We here hypothesized that 5-HT_2A_ downregulation and the normalization of 5-HT_2A_ signaling could contribute to the antidepressant-like effects of serotoninergic psychedelics. To test our hypothesis, we investigated whether the exposure of mice to swim stress altered 5-HT_2A_ receptor-mediated head twitch responses and 5-HT_2A_ receptor protein levels in the plasma membrane fractions of the medial prefrontal cortex (mPFC). Moreover, we investigated whether the synthetic serotoninergic psychedelic, DOI, altered forced-swim-test behavior in mice and 5-HT_2A_ receptor protein levels in the plasma membrane fractions of the mPFC.

## 2. Results

### 2.1. Head Twitch Response

We first investigated whether exposure to swim stress had long-lasting effects on 5-HT_2A_ receptor sensitivity, as measured by the head twitch response following the administration of DOI. We chose DOI for our experiments given that this compound is extensively characterized in rodents as a selective 5-HT_2_ receptor agonist with an approximately 4-fold higher affinity for 5-HT_2A_ compared to 5-HT_2C_ [92,93]. Moreover, previous studies have shown that the effects of DOI (in a dose of up to 2 mg/kg) on head twitching and other behaviors are completely abolished in 5-HT_2A_ receptor knockout mice. Head twitch behavior was observed immediately after drug administration [15,94,95].

**Figure 1:**
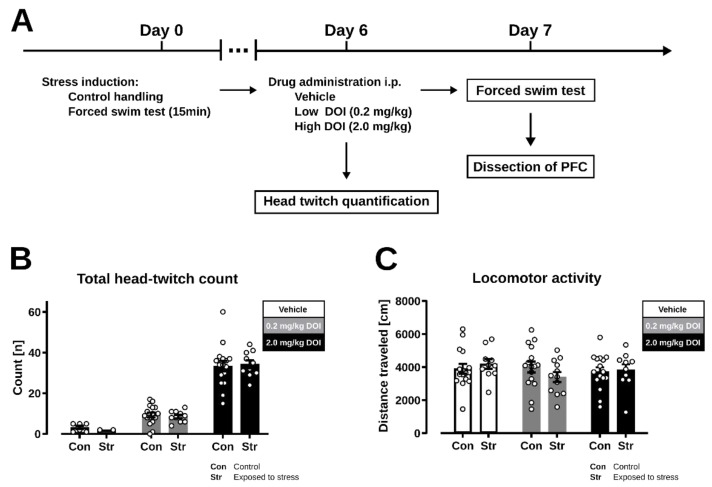
Effect of swim-stress exposure on 5-HT_2A_ receptor-mediated behavior in mice. (**A**) Experimental design. Mice were exposed to 15 min forced swim stress or stayed in a home cage on day 0. On day 6, mice received a single injection of a vehicle solution (1% DMSO in saline, Con: *n* = 16, Str: *n* = 12) or DOI (low dose: 0.2 mg/kg, Con: *n* = 16, Str: *n* = 10; high dose: 2.0 mg/kg, Con: *n* = 16, Str: *n* = 10). Behavior of mice was immediately recorded for locomotor activity and head twitch assessment. (**B**) Total head twitch count observed for 15 min after treatment. Head twitch scores for the vehicle groups were obtained and plotted for a limited number of mice and used as a visual reference. (**C**) Activity measured by distance traveled. Data are represented as mean values +/− SEM.

We found that DOI dose-dependently increased head twitch responses but the effect of DOI was not influenced by prior stress exposure (Figure 1B; treatment: F (1, 48) = 152.9, *p* < 0.0001; stress: F (1, 48) = 0.005, *p* = 0.95, interaction: F (1, 48) = 0.18; *p* = 0.67). The administration of DOI had no significant effect on the distance traveled by mice, irrespective of prior stress exposure (Figure 1C; treatment: F (2, 74) = 0.66, *p* = 0.52, stress: F (1, 74) = 0.08, *p* = 0.78, interaction F (2, 74) = 1.14, *p* = 0.33). Taken together, these findings indicate that prior exposure to swim stress had no long-lasting effects on the evaluated measure of 5-HT_2A_ receptor sensitivity.

### 2.2. Forced Swim Test

**Figure 2:**
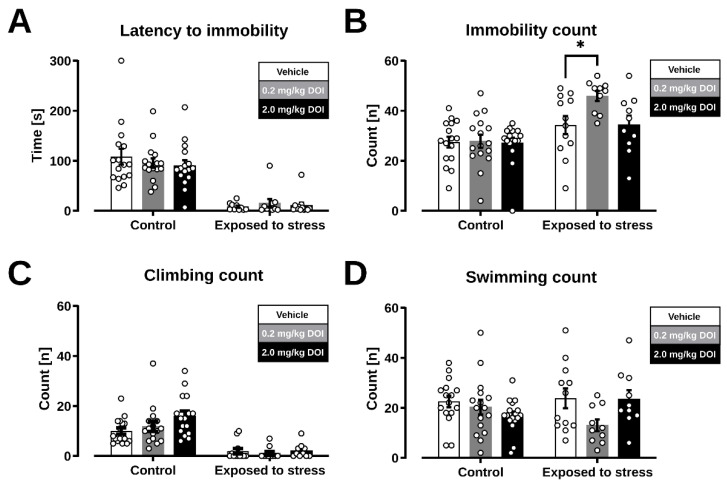
Effect of DOI on passive stress-coping behavior in the forced swim test in mice. (**A**) Latency to immobility. (**B**) Immobility count. (**C**) Climbing behavior. (**D**) Swimming behavior. Plots represent classification of 5 s intervals. Data are represented as mean values +/− SEM. * *p* < 0.05 vs. Veh group.

We hypothesized that the downregulation of 5-HT_2A_ receptors in the mPFC and the associated changes in 5-HT_2A_ signaling may contribute to the antidepressant-like effects of serotoninergic psychedelics. Mice received a single administration of DOI on day 6. The forced swim test was performed on day 7 to exclude the possible locomotor effects of the compound and to ensure sufficient time for 5-HT_2A_ receptor downregulation [32]. Prior exposure to swim stress significantly reduced the latency to immobility during the forced swim test as mice readily adopted a passive stress-coping strategy. However, we found no significant effects for treatment with DOI (Figure 2A; treatment: F (2, 74) = 0.24, *p* = 0.79, stress: F (1, 74) = 85.75, *p* < 0.0001; interaction: F (2, 74) = 0.52, *p* = 0.60). Previous swim-stress exposure also eliminated climbing behavior in mice, while DOI treatment had no significant effect (Figure 2C; treatment: F (2, 74) = 1.97, *p* = 0.15; stress: F (1, 74) = 64.91, *p* < 0.0001; interaction: F (2, 74) = 1.56, *p* = 0.22) and we found that prior exposure to swim stress significantly increased the time spent immobile in the forced swim test but also observed a significant effect for treatment with DOI (Figure 2B; treatment: F (2, 74) = 3.19, *p* = 0.047; stress: F (1, 74) = 22.94, *p* < 0.0001; interaction: F (2, 74) = 2.70, *p* = 0.07). Post-hoc analysis revealed that the lowest dose of DOI significantly increased immobility in mice previously exposed to swim stress (*p* = 0.01). During the analysis of swimming behavior, no significant effects of stress exposure or DOI treatment effects were observed (Figure 2D; treatment: F (2, 74) = 2.62, *p* = 0.08; stress: F (1, 74) = 0.008, *p* = 0.93; interaction: F (2, 74) = 2.90, *p* = 0.06).

### 2.3. 5-HT_2A_ Receptor Protein Levels

**Figure 3:**
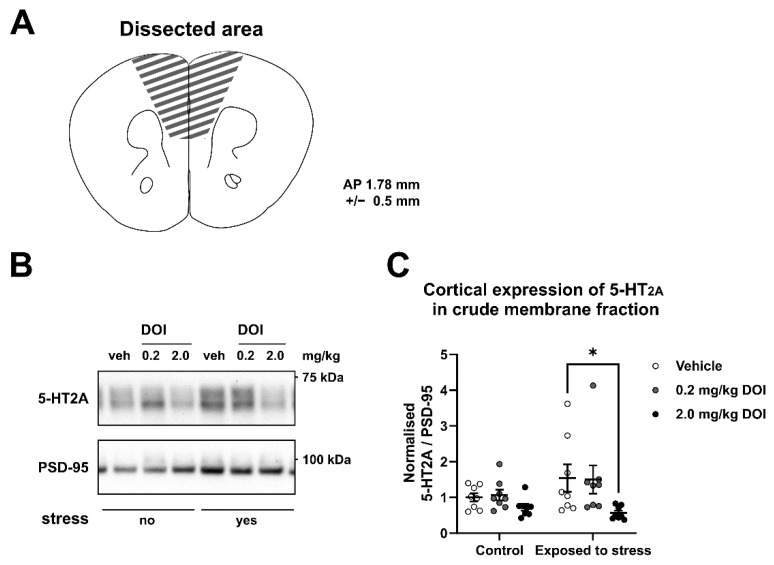
Effects of DOI administration on 5-HT_2A_/PSD-95 ratio in a crude membrane fraction of the mouse mPFC. (**A**) Representation of a target area. (**B**) Representative western blot results for the 5-HT_2A_ receptor and PSD-95 signal. (**C**) Quantification of 5-HT_2A_/PSD-95 signal ratio, normalized to control vehicle group. Data are represented as mean values +/− SEM. * *p* < 0.05 vs. Veh group.

Finally, we analyzed 5-HT_2A_ receptor levels in crude membrane fractions prepared from mPFC samples (Figure 3A). We used PSD-95 as a loading control given that this membrane-associated protein is critical for tethering 5-HT_2A_ receptors at the cell surface [35] (Figure 3B). Analysis of processed 5-HT_2A_/PSD-95 ratios showed no significant effects for prior stress exposure but a significant effect for treatment with DOI (Figure 3C; treatment: F (2, 42) = 4.593, *p* = 0.02; stress: F (1, 42) = 1.93, *p* = 0.17; interaction: F (2, 42) = 1.20, *p* = 0.31). Post-hoc analysis revealed that the administration of the highest dose of DOI induced a significant reduction in the 5-HT_2A_/PSD-95 ratio in mice previously exposed to stress (*p* = 0.01). Together, these results indicate that the membrane levels of 5-HT_2A_ receptors in stressed mice are significantly reduced one day after the administration of DOI. However, lowered 5-HT_2A_ receptor protein levels were not associated with changes in immobility in the forced swim test.

## 3. Discussion

We hypothesized that 5-HT_2A_ downregulation and the normalization of 5-HT_2A_ signaling could contribute to the antidepressant-like effects of serotoninergic psychedelics. While mice exposed to swim stress rapidly developed a passive stress-coping strategy, stress exposure did not lead to a higher sensitivity of 5-HT_2A_ receptors or increased membrane levels of these receptors in the mPFC. While the administration of DOI led to a pronounced decrease in 5-HT_2A_ receptor levels in the mPFC of mice previously exposed to stress, this was not associated with lower immobility in the forced swim test.

Brief stress exposure has been previously reported to induce a transient suppression of the 5-HT_2A_ receptor-mediated head twitch response [96] followed by a delayed and long-lasting increased head twitch [71]. This is associated with a stress-induced increase in 5-HT_2A_ receptor density in the cortex [70,71,72]. Likewise, repeated stress was shown to increase the 5-HT_2A_ receptor-mediated head twitch response [76,77] and the cortical density of the 5-HT_2A_ receptors [72,74,75,97]. However, some studies also found no significant effects of stress exposure on cortical 5-HT_2A_ receptor binding or 5-HT_2A_ receptor-mediated head twitch responses in rodents [85,86,87]. In our experiments, we observed a trend towards increased 5-HT_2A_ receptor levels in mPFC membrane fractions. However, this did not reach statistical significance. Similarly, we found no significant effects for swim stress on 5-HT_2A_ receptor-dependent head twitch responses following the administration of DOI. Taken together, it remains unclear whether and how stress exposure would lead to long-lasting changes in 5-HT_2A_ receptor levels in the rodent mPFC. Differences in the type of stressor, duration of stress exposure, time since stress exposure or technical differences related to the isolated brain region or to the method for the determination of 5-HT_2A_ receptor levels may contribute to the differences in the literature [88]. Moreover, a more complex role of 5-HT_2A_ in stress coping could be suspected. Indeed, 5-HT_2A_ receptor deficiency was recently shown to alter the metabolic and transcriptional but not behavioral consequences of chronic unpredictable mild stress [98].

The downregulation of 5-HT_2A_ receptors has been observed following the chronic administration of SSRI [36,37,38,39], and 5-HT_2A_ antagonism exerts antidepressant-like effects [41,42,43,44] or increases the efficacy of antidepressants [40,42,45,46,47]. We therefore hypothesized that the DOI-induced downregulation of 5-HT_2A_ receptors would be associated with an antidepressant-like response in the forced swim test. Importantly, the genetic ablation of 5-HT_2A_ receptors in mice does not induce an antidepressant-like response per se [99], but interferes with the antidepressant-like responses to SSRI [100]. DOI-induced desensitization and the internalization of 5-HT_2A_ receptors have been observed previously [16,32,101], and 5-HT_2A_ receptors present a cross-tolerance effect to psychedelics, related to the downregulation of these receptors independent of β-arrestin 2, a protein typically involved in the trafficking of 5-HT_2A_ receptors after its interaction with non-psychedelic agonists [16,32]. Interestingly, while non-psychedelic 5-HT_2A_ agonists (such as 5-HT) or inverse agonists (such as clozapine) were shown to elicit rapid internalization with recycling in approximately 2.5 h, the psychedelic agonist DOI induced slow internalization with recycling in approximately 7.5 h [101]. In addition, repeated administration with a non-psychedelic agonist of the 5-HT_2A_ receptor does not have an effect on the efficacy of LSD or DOI to induce a behavioral response [32]. These differences in downstream effects and recycling kinetics could be associated with variations in 5-HT_2A_ phosphorylation induced by non-psychedelic and psychedelic ligands [16]. Our results support the notion that DOI elicits the downregulation of 5-HT_2A_ receptors in the cortex for at least 24 h. Previous studies similarly found that the single and repeated administration of DOI resulted in functional desensitization and reduced 5-HT_2A_ receptor binding in rodents [31,32]. However, functional 5-HT_2A_ receptor desensitization does not necessarily correspond to decreased total 5-HT_2A_ protein levels but may reflect redistribution from the plasma membrane to the cytosol [31]. Given the time course of the observed effects on 5-HT_2A_ levels, it is possible that, beyond internalization, DOI will also affect 5-HT_2A_ receptor expression through transcriptional and translational mechanisms. Interestingly, we observed that DOI-induced 5-HT_2A_ receptor downregulation was more pronounced in mice that were previously exposed to swim stress. Similarly, SSRIs were previously shown to induce stronger 5HT2A receptor downregulation in isolation-reared mice [102]. Nevertheless, the functional consequences of reduced 5-HT_2A_ receptor levels in the mPFC remain unclear since in our study we found no association with reduced immobility in the forced swim test.

Previous studies have investigated the effects of serotoninergic psychedelics on stress coping in rodents [12,89,103,104,105,106,107]. One previous study found that a single administration of DOI did not have an acute effect on immobility behavior in the forced swim test in rats [42]. Similarly, the repeated administration of LSD did not induce antidepressant-like effects in non-stressed mice [104]. A single dose of psilocybin had no significant effect on immobility in the forced swim test in rats, when the test procedure followed 24 h after drug administration [107]. Moreover, single or repeated doses of psilocybin had no antidepressant-like effects in control rats or the Flinders Sensitive Line rat model of depression [103]. In contrast to these studies, both LSD and psilocybin were shown to induce delayed antidepressant-like effects in rats exposed to a forced swim test, up to 5 weeks after the drug administration [91]. In other studies, DOI reduced immobility when administered 24 h before the forced swim test in mice that were not previously exposed to swim stress [108] and the non-psychedelic ibogaine analog (tabernanthalog; TBG) reduced immobility when administered 24 h before the forced swim test in mice that were previously exposed to a single session of swim stress [106]. Moreover, the repeated administration of N,N-dimethyltryptamine (DMT) after swim-stress exposure and before the forced swim test significantly reduced immobility in rats [89]. Taken together, it remains uncertain why these discrepancies have been described in the literature. We propose that other models with extensive stress exposure, such as the chronic unpredictable mild stress paradigm, may be more informative when studying the antidepressant-like effects of psychedelics.

It is tempting to speculate that the antidepressant-like effects of psychedelics are dependent on the duration and type of prior stress exposure, the timing between stress exposure and psychedelic administration and the dose and duration of the psychedelic administration. In this context, our observation of increased immobility after a low dose of DOI in mice that were previously exposed to swim stress remains puzzling. This effect was not associated with the increased sensitivity of the 5-HT_2A_ receptor at the time of DOI administration, nor did it significantly alter 5-HT_2A_ receptor levels in the mPFC at the time of the forced swim test. The notion that this effect of the low dose of DOI was only observed in mice previously exposed to swim stress suggests a role of the memory in acquired stress-coping behaviors. Indeed, 5-HT_2A_ receptors appear to play a role in associative learning and memory systems [109,110,111]. While in humans serotoninergic psychedelics produce dose-dependent increasing impairments in spatial memory task performance, they also stimulate the recall of autobiographical memories and increase the vividness of these memories [112]. However, few studies have investigated the long-term effects. One study found that a low dose of LSD improves measures for visuospatial memory 24 h after administration [113]. In our experiments, increased immobility 24 h after the administration of DOI may thus reflect better memory recall for the previous swim-stress exposure and a more robust expression of the corresponding passive stress-coping mechanism. However, given that 5-HT_2A_ receptors are necessary for episodic memory recall and reconsolidation [110,111], and that the highest dose of DOI decreased mPFC 5-HT_2A_ receptors 24 h later, this may have resulted in poorer memory recall for the previous swim-stress exposure.

It remains unclear whether the 5-HT_2A_ receptor is involved in the previously reported antidepressant-like effects of psychedelics [12]. Indeed, the 5-HT_2A_/5-HT_2C_ antagonist ketanserin (4 mg/kg) blocked the antidepressant-like effects of the ibogaine analog TBG in a forced swim test paradigm similar to the one used in our study [106], while a slightly lower dose of ketanserin (2 mg/kg) did not reverse the antidepressant-like effects of psilocybin in a chronic multimodal stress paradigm [12]. Experiments in 5-HT_2A_ receptor knockout mice should further resolve this issue, and further demonstrate that the antidepressant-like effects of serotoninergic psychedelics are indeed mediated by 5-HT_2A_ receptors and do not involve any of the other receptors for which serotoninergic psychedelics show affinity, such as 5-HT_2C_ or 5-HT_1A_ [4].

Interestingly, the activation of 5-HT_2C_ receptors appears to oppose the behavioral effects of 5-HT_2A_ activation. Indeed, 5-HT_2C_ receptor agonists do not produce head twitch responses but dose-dependently suppress DOI-induced head twitch responses in mice [114,115]. Similarly, while 5-HT_2A_ activation increases locomotor activity and decreases anxiety, 5-HT_2C_ agonists produce hypolocomotion [94,115] and increase anxiety [116,117]. Moreover, 5-HT_2C_ overexpressing mice show hypolocomotion and increased anxiety [118], whereas 5-HT_2C_ knockout mice show increased exploratory activity and decreased anxiety [119]. Importantly, heteromerization has been described for 5-HT_2A_ and 5-HT_2C_ receptors. The notion that the binding properties of the 5-HT_2A_ protomer are influenced by 5-HT_2C_ receptors suggests an allosteric mechanism [120]. This is indeed also supported by the observation that the DOI-induced suppression of dorsal raphe firing is abolished in 5-HT_2A_ knockout mice [40] but also attenuated by the exogenous overexpression of an inactive form of the 5-HT_2C_ receptor in the locus coeruleus [121]. Whereas 5-HT_2A_ is the preferential target of lower doses of DOI, 5-HT_2A_ signaling can clearly be influenced by 5-HT_2C_ receptors, either by the direct binding of psychedelics to these receptors, or through heteromerization and allosteric mechanisms. This implies that altered 5-HT_2C_ receptor levels, induced by the administration of psychedelics such as DOI, may also affect behavioral outcomes. Future studies should therefore consider the interplay between 5-HT_2A_ and 5-HT_2C_ receptors more carefully.

Finally, while the 5-HT_1A_ receptor is not a high-affinity target for DOI, it has been demonstrated to have a modulatory role in the effects of other serotoninergic psychedelics such as psilocybin and LSD [122,123]. In this context, LSD was shown to induce a rebalancing 5-HT_2A_/5-HT_1A_ signaling, with a decrease in 5-HT_2A_ signaling and an increase in 5-HT_1A_ signaling in the hippocampus of the bulbectomy rat model [124]. Moreover, increased cortical spinogenesis and an enhancement of 5-HT neurotransmission following repeated LSD administration in stress-exposed mice were associated with 5-HT_1A_ receptor desensitization in dorsal raphe 5-HT neurons [104]. Indeed, psychedelics can have a pervasive effect on 5-HT signaling, through presynaptic and postsynaptic mechanisms [109,125], but the pharmacological mechanisms through which they exert their antidepressant-like effects in rodents are not clear. In addition, to what extent these observations in rodents can be translated to humans remains unclear. One important limitation of our study is that our conclusions are restricted to behavioral observations in the forced swim test. It is clear that the forced swim test does not replicate the broad spectrum of a depression-like phenotype [126] and may lack predictive validity [127,128]. Examining the effects of psychedelics on the performance of rodents in tests measuring appetitive behaviors, such as the female urine test [12] and the sucrose preference test [129], could provide a complementary perspective. This may be particularly true for psychedelics, where the human psychedelic experience may be difficult to model in rodents, and where the evaluation of parameters that can also be observed in humans, such as functional connectivity in brain networks [130] may hold better translational value.

## 4. Materials and Methods

### 4.1. Animals

All experiments were carried out on male C57BL/6JRj mice (Janvier, Le Genest-Saint-Isle, France). Mice were 8–12 weeks old at the time of experiments and were group-housed (5 per cage; 425 × 276 × 153 mm; 1290D Eurostandard Type III cages, Tecniplast, Buguggiate, Italy) in standard laboratory conditions with a 12/12 h day-night cycle, and controlled temperature (20–24 °C) and humidity (30–60%). Food (A04, Safe Diets, Augy, France) and water were provided ad libitum. Cages were enriched with nesting material, gnawing sticks and a Mouse-House shelter (Tecniplast, Buguggiate, Italy). Mice were habituated to the animal facility at least one week prior to further manipulation. Before the first behavioral test, mice were habituated to handling by the male experimenter for approximately two minutes per day on three consecutive days. The behavioral experiments were carried out between 8:30 and 14:30.

### 4.2. Drugs and Administration

Stock solutions of the 5-HT_2A_/5-HT_2C_ receptor agonist 2,5-dimethoxy-4-iodoamphetamine hydrochloride (DOI, Tocris Bioscience, Bristol, UK) were prepared in 99.9% dimethylsulfoxide (DMSO; Sigma-Aldrich Chemicals, Darmstadt, Germany) and stored at −20 °C. Working solutions were prepared by diluting the stock solution in sterile saline (0.9% NaCl, Baxter, Brussels, Belgium) the day before the administration and stored overnight at +4 °C until use. DOI was diluted to the final concentration in an intraperitoneal (i.p.) injection volume of 10 mL/kg. The working solutions contained up to 5% V/V DMSO in sterile saline. Control mice received 10 mL/kg of 5% V/V DMSO in sterile saline. The low (0.2 mg/kg) and high (2.0 mg/kg) doses were selected based on preliminary data and the previous literature [95,131]. Mice received drug injections on day 6 of the experiment, one day before a forced swim test procedure and brain tissue extraction (Figure 1A).

### 4.3. Head Twitch Response

The administration of DOI or other psychedelics induces head twitch responses in mice [132]. This stereotypical behavior is driven by the activation of 5-HT_2A_ receptors and can be used as an indirect in vivo measure of the sensitivity of these receptors [71,133]. To evaluate head twitch responses, mice were placed in a single housing cage (268 × 215 × 141 mm; 1264C Eurostandard Type II cages, Tecniplast, Buguggiate, Italy) in the experimental room for a habituation period of 60 min. The testing cage had fresh bedding material mixed in with the bedding material of the homecage of the tested mouse. After habituation, mice were injected with vehicle or DOI and their behavior was recorded by a webcam placed 50 cm above the cage and registered in MP4 format (Debut v 2.02_,_ NCH software, Greenwood, CA, USA). Mouse behavior was recorded for a duration of 15 min following drug administration. The activity of mice, measured by their velocity and distance traveled, was analyzed with Ethovision XT (Noldus, Wageningen, The Netherlands) and an observer blinded to treatment scored the number of head twitch responses manually. Although head twitch behavior was only expected in mice treated with DOI, we also plotted the head twitch responses of mice treated with vehicle as a reference (*n* = 6 in non-stressed group; *n* = 2 in stress exposed group).

### 4.4. Locomotor Activity

The locomotor activity of mice was extracted from video recordings obtained during the head twitch observation, where each mouse was observed in single housing cage (268 × 215 × 141 mm; 1264C Eurostandard Type II cages, Tecniplast, Buguggiate, Italy) with bedding from their home cage mixed in. Each mouse was monitored for 15 min by a video tracking system (Ethovision XT, Noldus, Wageningen, The Netherlands). The activity was measured as the distance traveled following the calibration of the video tracking software.

### 4.5. Forced Swim Test

When exposed to inescapable swim stress, mice develop a passive stress coping strategy (immobility), that is typically interpreted as an indicator of depressive-like behavior [134,135,136]. We carried out a modified version of the forced swim test (day 0) as previously described [71]. Mice were pre-exposed to 15 min swim stress and were subjected to a 5 min forced swim test 6 days later (on day 7). Additional control mice were included that were not exposed to 15 min swim stress but were handled and subjected to a 5 min forced swim test 6 days later. During the first exposure to swim stress and the subsequent forced swim test, mice were placed in a brightly illuminated (400 lux) cylinder glass tank (diameter: 16 cm, height: 24 cm) filled with tap water (25 ± 1 °C, 17 cm deep). Mice were closely observed during the procedures and their behavior was recorded by a webcam placed 30 cm in front of the glass tank and registered in MPG format (Debut v 2.02_,_ NCH software, Greenwood, CA, USA). After each session, mice were removed from the glass tank, carefully dried with a paper towel and placed in an externally warmed recovery cage for at least 10 min after which they were returned to their home cage. An observer that was blinded to treatment analyzed the stress-coping behavior during the 5 min forced swim test by classifying the most predominant behavior per 5 sec interval. Climbing was scored when mice took a vertical body position and its paws broke the surface of the water, swimming was scored when the animal had a horizontal body position and travelled at least a diameter of the cylinder, immobility was scored otherwise. The analysis was performed on the counts of predominant behaviors.

### 4.6. Western Blot

The protein levels of 5-HT_2A_ receptors were analyzed by Western blot. Within 5 min after the forced swim test, mice were sacrificed by neck dislocation, and the medial part of the prefrontal cortex (mPFC; anterior-posterior 1.5 +/− 0.5 mm, according to the stereotactic brain atlas) was dissected, snap-frozen in 2-methylbutane on dry ice (Sigma-Aldrich, Darmstadt, Germany) and stored at −80 °C. Crude membrane fractions were prepared as follows: pre-chilled lysis buffer (0.32 M sucrose in HEPES 5 mM, pH 7.4; Sigma-Aldirch, Darmstadt, Germany) containing ethylenediaminetetraacetic acid (EDTA; (ThermoFisher, Bremen, Germany), HALT protease inhibitors (ThermoFisher, Bremen, Germany), and phosphatase inhibitor cocktail II (ThermoFisher, Bremen, Germany) was added to the frozen tissue at a volume of 10 μL per mg of tissue. Samples were homogenized with a pestle connected to a drill, for 20 s. Homogenized samples were cleared at 1000 *g* to remove nuclei and large debris. The resulting supernatants were concentrated twice at 12,000 *g* for 20 min to obtain a crude membrane fraction, each time resuspended in a 5 mM HEPES pH 7.4 buffer. All preparation steps were performed at +4 °C, and prepared samples were aliquoted and stored at −80 °C.

To analyze 48 samples (*n* = 8 per experimental group), the full experiment was loaded onto two Criterion Bis-Tris 10% gels (Bio-Rad) that were processed in parallel. One aliquot of each sample was used to calculate protein concentration with a Pierce BCA assay (ThermoFisher, Bremen, Germany). Sample volumes corresponding to 15 μg of protein were mixed with XT loading buffer (Bio-Rad, Temse, Belgium) and XT reducing agent (Bio-Rad) and loaded into gels. Precision Plus Protein Dual Color ladder (Bio-Rad, Temse, Belgium) was loaded into a middle well. After electrophoresis was performed in MES XT running buffer (Bio-Rad, Temse, Belgium), the resolved samples were wet-transferred to PVDF membranes (Bio-Rad, Temse, Belgium). The protocol was optimized for incubation in 50 mL falcon tubes on a rotator. The membrane was cut into two pieces, along the ladder in order to fit into the tube. Membrane pieces were then processed in parallel as follows. The pieces were blocked with 4% bovine serum albumin (BSA) in tris-buffered saline with 0.1% Tween-20 (TBST; Tris from Bio-Rad, Belgium, Tween-20 obtained from Sigma-Aldrich, Bremen, Germany) for 60 min at room temperature. The membrane pieces were then incubated in TBST with anti-5-HT_2A_ receptor rabbit antibodies (1:500; Immunostar, Hudson, WI, USA) overnight on a rotator. The next morning, membrane pieces were rinsed and washed with TBST three times and incubated with HRP-conjugated secondary antibodies (1:12,500; Cell Signaling Technology, Leiden, The Netherlands) in TBST, for 60 min at room temperature on a rotator. Membrane pieces were then rinsed and washed with TBST five times for 10 min before incubating with SignalFire ECL Plus Reagent (Cell Signaling Technology, Leiden, The Netherlands), for 1 min. The images were acquired with ChemiDocMP (Bio-Rad, Temse, Belgium), in signal accumulation mode. After obtaining the signal for the targeted protein, membrane pieces were stripped with a 2.2 pH mild stripping buffer containing 0.0035 M of sodium dodecyl sulfate (SDS; Sigma Aldrich, Bremen, Germany), 0.2M glycine (Bio-Rad, Temse, Belgium) and 1% Tween-20. Membrane pieces were then incubated overnight with rabbit antibodies targeted at PSD-95 (1:2000; Cell Signaling Technology, Leiden, The Netherlands). The rest of the steps required for detection took place as described above, using a secondary antibody concentration 1:30,000. All used solutions were prepared using milliQ water. The intensities of the 5-HT_2A_ receptor signal and the PSD-95 signals were measured with ImageJ version 1.5.3 (National Institutes of Health, Bethesda, Maryland, USA) *Gel Analyze* plugin from Fiji distribution package [137]. For each sample, the 5-HT_2A_/PSD-95 ratio was determined and normalized to the average ratio obtained for the non-stressed vehicle samples on each membrane piece.

### 4.7. Statistics

Statistical analysis was performed using Graphpad Prism software 9.1.2 (Graphpad Software, San Diego, CA, USA). Values are expressed as mean ± s.e.m and α was set at 0.05. Two-way ANOVA was performed using treatment and prior exposure to swim stress as independent factors. Post-hoc comparisons were adjusted with Bonferroni’s multiple comparison test.

## 5. Conclusions

Taken together, our data do not show significant effects of swim-stress exposure on 5-HT_2A_ receptor sensitivity or 5-HT_2A_ receptor protein levels in the mPFC of mice. While the administration of the serotoninergic psychedelic DOI induced a significant reduction in 5-HT_2A_ receptors in the mPFC of mice previously exposed to swim stress, this was not associated with an antidepressant-like effect, measured as reduced immobility in the forced swim test. We suggest that further experiments aiming to describe the effects of serotoninergic psychedelics on stress coping should consider utilizing testing procedures that are not contingent on stress induction to avoid potential bias resulting from 5-HT_2A_ receptor involvement in the memory processes [111,138,139,140], and to explore how psychedelics affect different domains challenged by depression, such as reward-driven behaviors. As studies exploring the long-term effects of psychedelics involving neuroplasticity and gene expression are currently gaining traction [108,141,142,143], it would be interesting to explore the interaction of these processes with behavioral interventions.

## Data Availability

The data presented in this study are available from the corresponding author on request.
